# Heat and Mass Transfer Analysis of MHD Nanofluid Flow with Radiative Heat Effects in the Presence of Spherical Au-Metallic Nanoparticles

**DOI:** 10.1186/s11671-016-1692-2

**Published:** 2016-10-24

**Authors:** M. Zubair Akbar Qureshi, Qammar Rubbab, Saadia Irshad, Salman Ahmad, M. Aqeel

**Affiliations:** 1 Department of Computer Science, Air University, Multan Campus, Islamabad, Pakistan; 2 Department of Management Sciences, Air University, Multan Campus, Islamabad, Pakistan; 3 Department of Applied Mathematics and Statistics, Institute of Space Technology, Islamabad, Pakistan

**Keywords:** Thermal radiation effects, Au-metallic nanoparticles, Viscous dissipation, Wall expansion ratio

## Abstract

Energy generation is currently a serious concern in the progress of human civilization. In this regard, solar energy is considered as a significant source of renewable energy. The purpose of the study is to establish a thermal energy model in the presence of spherical Au-metallic nanoparticles. It is numerical work which studies unsteady magnetohydrodynamic (MHD) nanofluid flow through porous disks with heat and mass transfer aspects. Shaped factor of nanoparticles is investigated using small values of the permeable Reynolds number. In order to scrutinize variation of thermal radiation effects, a dimensionless Brinkman number is introduced. The results point out that heat transfer significantly escalates with the increase of Brinkman number. Partial differential equations that govern this study are reduced into nonlinear ordinary differential equations by means of similarity transformations. Then using a shooting technique, a numerical solution of these equations is constructed. Radiative effects on temperature and mass concentration are quite opposite. Heat transfer increases in the presence of spherical Au-metallic nanoparticles.

## Background

Today, solar thermal systems with nanoparticles have become a new area of investigation. Further thermal radiative transport has notable significance in several applications in the field of engineering such as solar power collectors, astrophysical flows, large open water reservoirs, cooling and heating chambers, and various other industrialized and environmental developments. Nanoparticles have an ability to absorb incident radiations. Bakier [1] explored how thermal radiation affects mixed convection from a vertical surface in a porous medium. Damseh [2] looked at effects of radiation heat transfer and transverse magnetic field in order to perform numerical analysis of magnetohydrodynamics-mixed convection. Hossain and Takhar [3] analyzed how radiation influences forced and free convection flow on issues related to heat transfer. In a study, Zahmatkesh [4] explored that temperature is almost uniformly distributed in the vertical sections inside an enclosure as a result of thermal radiation. The findings of this study concluded that the streamlines are almost parallel along the vertical walls. An analysis of thermal radiation in forced and free convection flow on an inclined flat surface was carried out by Moradi et al. [5]. In the same vein, Pal and Mondal [6] examined results of radiation on forced and free convection on a vertical plate set in a porous medium having variable porosity. Hayat et al. [7] extended thermal radiation results in magnetohydrodynamic (MHD) steady nanofluid flow through a rotating disk.

Nanofluids are a new dynamic sub-class of nanotechnology. This is the reason why the majority of scientists and researchers are persistently attempting to take a shot at novel elements of nanotechnology. Das and Choi [8] named the amalgamation of these particulate matters of particle size in the order of nanometers as a “nanofluid.” Nano-particulate suspension in a base fluid makes it superior and finer in terms of heat transfer compared to conventional fluids. Abrasion-related properties of nanofluids are found to be excellent over traditional fluid-solid mixtures. Metallic nanoparticles have vast applications in the ambit of nanosciences. Nanofluids with metallic nanoparticles have a lot of useful applications especially in the biological sciences. The photothermal metallic nanoblade is another novel methodology for delivering highly concentrated material into mammalian cells. Cryosurgery is used to destroy undesired tissues with penetration of metallic nanoparticles into the target tissues. Gold nanoparticles are the finest and most efficient drug-carrying molecules. The injection/suction factor with relaxing/contracting porous orthogonally moving disks in well-established flows is regarded as an important area of study in fluid mechanics. This area of study has attracted significant applications in engineering sciences, for example, crystal growth procedures, computer storage equipment, rotating machineries, viscometers, heat and mass exchangers, and lubricants [9–13]. Ashraf et al. [14] discussed non-Newtonian fluid flow in orthogonally moving coaxial porous and non-porous disks. Kashif et al. [15] conducted a ground-breaking study of nanofluid flow due to orthogonally porous moving disks. The core principles of magnetohydrodynamics flow are particularly used in spacecraft propulsion, plasma accelerators for ion thrusters, light ion beam, powered inertial confinement, MHD generators, pumps, bearing, and boundary layer flow in aerodynamics. Nikiforov [16] performed a seminal study on MHD flow. Various other analysts have also emphasized this idea, and points of interest are explored in various studies, for example, Hatami et al. [17, 18], Sheikholeslami et al. [19–26], Hayat et al. [27–29], Rashidi et al. [30], Mehrez et al. [31], Mabood et al. [32], Abbasi et al. [33], and Shehzad et al. [34].

Thermal radiation with viscous dissipation effects in nanofluid flow between porous orthogonally moving disks has to the best of our knowledge not been deliberated. Spherical Au-metallic nanoparticles are considered with a Hamilton–Crosser thermal conductivity model. In order to determine possible anomalous heat transfer enhancement related to spherical Au-metallic nanoparticles, volume fraction, velocity, temperature, and mass transport equations for permeability, Reynolds number and relaxing/contracting parameters are investigated. Mathematical modeling is undertaken and numerical results are constructed using a shooting method.

## Methods

**Fig. 1:**
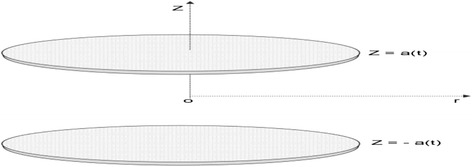
Physical geometry

Consider two-dimensional MHD unsteady laminar incompressible nanofluid flowing in porous coaxial disks of width 2*a*(*t*) with viscous dissipation and thermal radiation effects. Compared to the force field, the induced magnetic field is believed to be insignificant. It is assumed that there is no applied polarization. Water is taken as the base fluid. Thermal equilibrium exists between base fluid and nanoparticles. The thermophysical properties are shown in Table 1. Permeability of the disks is similar, with time dependent rate *a* ' (*t*) (shown in Fig. 1). Thermal conductivity is the most vital thermophysical property that influences nanofluid heat transfer rate. In order to explore efficient thermal conductivity of nanofluids, various theoretical models are currently available. Numerous theoretical studies are discussed in the literature to envisage appropriate models for effective viscosity along with thermal conductivity of nanofluids. The Hamilton–Crosser (H-C) model is the most common model for effective thermal conductivity of nanofluids and is given by [35]

**Table 1:** Thermophysical properties of water and metallic nanoparticles

	*ρ*(*Kgm* ^− 3^)	*C*_*p*_(*JKg* ^− 1^ *K* ^− 1^)	*K*(*Wm* ^− 1^ *K* ^− 1^)
H_2_O	997.1	4179	0.613
Au (metallic)	19,300	1290	318

1 $$ {k}_{\mathrm{nf}}={k}_{\mathrm{f}}\left[\frac{\left({k}_{\mathrm{s}}+\left(n-1\right){k}_{\mathrm{f}}\right)-\left(n-1\right)\phi \left({k}_{\mathrm{f}}-{k}_{\mathrm{s}}\right)}{\left({k}_{\mathrm{s}}+\left(n-1\right){k}_{\mathrm{f}}\right)+\phi \left({k}_{\mathrm{f}}-{k}_{\mathrm{s}}\right)}\right]. $$

Here *k*_nf_ denotes effective thermal conductivity of the nanofluid, *k*_f_ thermal conductivity of the continuous phase, *ϕ* the nanoparticles volume fraction, and “*n*” the shape factor for nanoparticles given by $\frac{3}{\psi }$ where *ψ* is the sphericity of the nanoparticles and determined by the shape of the nanoparticles [36, 37]. For spherical nanoparticles *ψ* = 1 or *n* = 3 and for cylindrical nanoparticles *ψ* = 0.5 or *n* = 6.

The geometry of the problem recommends that a cylindrical coordinate system may be selected with the origin at the center of the two disks. We take *u* and *w* as velocity components in the *r* and *z* directions, respectively. The governing equations for the problem, taking into account effects of thermal radiation and viscous dissipation, are as follows:

2 $$ \frac{\partial u}{\partial r}+\frac{u}{r}+\frac{\partial w}{\partial z}\kern0.5em =\kern0.5em 0, $$

3 $$ \frac{\partial u}{\partial t} + u\;\frac{\partial u}{\partial r}+w\;\frac{\partial u}{\partial z} = -\frac{1}{\rho_{\mathrm{nf}}}\frac{\partial p}{\partial r}+{\upsilon}_{\mathrm{nf}}\left(\frac{\partial^2u}{\partial {r}^2}+\frac{1}{r}\frac{\partial u}{\partial r}\kern0.5em -\frac{u}{r^2}+\frac{\partial^2u}{\partial {z}^2}\right)-\frac{\sigma_{\mathrm{e}}{B}_0^2}{\rho_{\mathrm{nf}}}u, $$

4 $$ \frac{\partial w}{\partial t} + u\kern0.24em \frac{\partial w}{\partial r} + w\kern0.24em \frac{\partial w}{\partial z} = -\frac{1}{\rho_{\mathrm{nf}}}\;\frac{\partial p}{\partial z}+{\upsilon}_{\mathrm{nf}}\;\left(\frac{\partial^2w}{\partial {r}^2} + \frac{1}{r}\kern0.24em \frac{\partial w}{\partial r} + \kern0.36em \frac{\partial^2w}{\partial {z}^2}\right)-\frac{\sigma_{\mathrm{e}}{B}_0^2}{\rho_{\mathrm{nf}}}v, $$

5 $$ \frac{\partial T}{\partial t} + u\kern0.24em \frac{\partial T}{\partial r} + w\kern0.24em \frac{\partial T}{\partial z} = {\alpha}_{\mathrm{nf}}\;\left(\frac{\partial^2T}{\partial {r}^2}+\frac{1}{r}\frac{\partial T}{\partial r}+\frac{\partial^2T}{\partial {z}^2}\right)+\frac{\mu_{\mathrm{nf}}}{{\left(\rho {c}_p\right)}_{\mathrm{nf}}}\;{\left(\frac{\partial u}{\partial z}\right)}^2-\frac{1}{{\left(\rho {c}_{\mathrm{p}}\right)}_{\mathrm{nf}}}\left(\frac{\partial {q}_{\mathrm{r}}}{\partial z}\right), $$

6 $$ \frac{\partial C}{\partial t} + u\kern0.24em \frac{\partial C}{\partial r} + w\kern0.24em \frac{\partial C}{\partial z} = D\;\left(\frac{\partial^2C}{\partial {r}^2}+\frac{1}{r}\frac{\partial C}{\partial r}+\frac{\partial^2C}{\partial {z}^2}\right), $$

where *σ*_e_ is the electrical conductivity, *B*_0_ is the strength of the magnetic field, *p* is the pressure, *T* is the temperature, *C* is the mass concentration, *D* is the mass diffusion coefficient, *α*_nf_ is the thermal diffusivity, *ρ*_nf_ is the density, and *υ*_nf_ is the kinematics viscosity of the nanofluid, are given by

7 $$ \begin{array}{l}{\upsilon}_{\mathrm{nf}}=\frac{\mu_{\mathrm{nf}}}{\rho_{\mathrm{nf}}},\;{\mu}_{\mathrm{nf}}=\frac{\mu_{\mathrm{f}}}{{\left(1-\phi \right)}^{2.5}},\;{\rho}_{\mathrm{nf}}=\left(1-\phi \right){\rho}_{\mathrm{f}}+\phi {\rho}_{\mathrm{s}},\;{\alpha}_{\mathrm{nf}}=\frac{k_{\mathrm{nf}}}{{\left(\rho {c}_{\mathrm{p}}\right)}_{\mathrm{nf}}},\\ {}{\left(\rho {c}_{\mathrm{p}}\right)}_{\mathrm{nf}}=\left(1-\phi \right)\;{\left(\rho {c}_{\mathrm{p}}\right)}_{\mathrm{f}}+\phi\;{\left(\rho {c}_{\mathrm{p}}\right)}_{\mathrm{s}},\;\end{array} $$

where *ρ*_s_ and *ρ*_f_ are, respectively, the densities of the solid fractions and fluid and (*ρc*_p_)_nf_ is the heat capacitance of the nanofluid. The boundary conditions are

8 $$ \begin{array}{l}u=0;v=-A{a}^{\prime }(t),\;\mathrm{at}\;z=-a(t)\;\mathrm{when}\;T={T}_1\;\mathrm{and}\;C={C}_1,\\ {}u=0;v=A{a}^{\prime }(t),\;\mathrm{at}\;z=a(t)\kern0.48em \mathrm{when}\;T={T}_1\;\mathrm{and}\;C={C}_1.\end{array} $$

Here, *A* is a measure of the disk permeability and the dash denotes derivative w.r.t. time *t*.

Using the Rosseland approximation for radiation, the radiative heat flux is

9 $$ {q}_{\mathrm{r}}=\frac{-4{\sigma}_{\mathrm{sB}}}{3{m}_0}\left(\frac{\partial {T}^4}{\partial z}\right), $$

where *σ*_sB_ is the Stefan-Boltzman constant and *m*_0_ is the mean absorption coefficient. Assume that difference in temperature within the flow is such that T^4^ can be expressed as a linear combination of temperature. Now, expand T^4^ in Taylor series about T_2_ as follows:

10 $$ {T}^4={T_2}^4+4{T_2}^3\left(T-{T}_2\right)+6{T_2}^2{\left(T-{T}_2\right)}^2+\dots $$

Neglect higher order terms beyond the first degree (*T* − *T*_2_) as follows:

11 $$ {T}^4\cong -3{T_2}^4+4{T_2}^3T. $$

By substituting Eq. (11) into Eq. (9) we obtain:

12 $$ \frac{\partial {q}_{\mathrm{r}}}{\partial z}=\frac{-16{\sigma}_{\mathrm{sB}}{T_2}^3}{3{m}_0}\left(\frac{\partial^2T}{\partial {z}^2}\right). $$

Now using Eq. (12) in Eq. (5), we obtain

13 $$ \frac{\partial T}{\partial t} + u\kern0.24em \frac{\partial T}{\partial r} + w\kern0.24em \frac{\partial T}{\partial z} = {\alpha}_{\mathrm{nf}}\;\left(\frac{\partial^2T}{\partial {r}^2}+\frac{1}{r}\frac{\partial T}{\partial r}+\frac{\partial^2T}{\partial {z}^2}\right)+\frac{\mu_{\mathrm{nf}}}{{\left(\rho {c}_{\mathrm{p}}\right)}_{\mathrm{nf}}}\;{\left(\frac{\partial u}{\partial z}\right)}^2+\frac{1}{{\left(\rho {c}_{\mathrm{p}}\right)}_{\mathrm{nf}}}\frac{16{\sigma}_{\mathrm{sB}}{T_2}^3}{3{m}_0}\left(\frac{\partial^2T}{\partial {z}^2}\right)\;. $$

After removing the pressure term from the governing equations, we introduce the following similarity transformation:

14 $$ \eta =z{a}^{-1},\;u=-r{\nu}_{\mathrm{f}}{a}^{-2}\;{F}_{\eta}\;\left(\eta, t\right),\;w=2{\nu}_{\mathrm{f}}{a}^{-1}F\;\left(\eta, t\right),\;\theta \left(\eta \right)=\frac{T-{T}_2}{T_1-{T}_2},\chi \left(\eta \right)=\frac{C-{C}_2}{C_1-{C}_2}. $$

The dimensions of *ν*_f_ are [*L* ^2^ *T* ^− 1^], those of both *u* and *w* are [*LT* ^− 1^], and finally [*L*] is the dimension of each of *a* and *r*, which when used in Eq. (14), give $F\left(=\frac{aw}{2{\nu}_{\mathrm{f}}}\right)$ and ${F}_{\eta}\left(=-\frac{a^2u}{r{\nu}_{\mathrm{f}}}\right)$ as the two dimensionless velocities in the axial and radial directions, respectively, between the porous disks. On the other hand, *θ*(*η*) and *χ*(*η*) being the ratio of two quantities having the same units is also dimensionless.

The transformation given in Eq. (14) leads to:

15 $$ \frac{\upsilon_{\mathrm{nf}}}{\upsilon_{\mathrm{f}}}{F}_{\eta \eta \eta \eta}+\alpha \left(3{F}_{\eta \eta }+\eta {F}_{\eta \eta \eta}\right)-2F{F}_{\eta \eta \eta }-\frac{a^2}{\upsilon_{\mathrm{f}}}{F}_{\eta\;\eta\;t}-\frac{\rho_{\mathrm{f}}}{\rho_{\mathrm{nf}}}M{F}_{\eta \eta }=0, $$

16 $$ \left(1+\left(4/3\right)Tr\right){\theta}_{\eta \eta }+\frac{\upsilon_{\mathrm{f}}}{\alpha_{\mathrm{nf}}}\left(\eta \alpha -2F\right){\theta}_{\eta }+\left({\left(1-\phi \right)}^{-2.5}{F_{\eta \eta}}^2\right){E}_{\mathrm{c}}\;{P}_{\mathrm{r}}\;\left(\frac{k_{\mathrm{f}}}{k_{\mathrm{nf}}}\right)-\frac{a^2}{\alpha_{\mathrm{nf}}}{\theta}_{\mathrm{t}}=0, $$

17 $$ \frac{D}{\upsilon_{\mathrm{f}}}{\chi}_{\eta \eta }+\left(\eta \alpha -2F\right){\chi}_{\eta }-{a}^2{\chi}_{\mathrm{t}}=0, $$

with boundary conditions:

18 $$ \begin{array}{l}F=-\mathrm{R}\mathrm{e};\;{F}_{\eta }=0,\;\mathrm{at}\;\eta =-1\;\mathrm{when}\;\theta =1\;\mathrm{and}\;\chi =1,\\ {}F=\mathrm{R}\mathrm{e};\;{F}_{\eta }=0,\;\mathrm{at}\;\eta =1\;\mathrm{when}\;\theta =0\;\mathrm{and}\;\chi =0.\end{array} $$

Here *T*_1_ and *T*_2_ (with*T*_1_ > *T*_2_) are the fixed temperatures of the lower and upper disks, respectively, $\alpha =\frac{a\;{a}^{\prime }(t)}{\upsilon_{\mathrm{f}}}$ is the wall expansion ratio, $\mathrm{R}\mathrm{e}=\frac{Aa\;{a}^{\prime }}{2{\upsilon}_{\mathrm{f}}}$ is the permeability Reynolds number, $M=\frac{\sigma_{\mathrm{e}}{B}_0^2{a}^2}{\mu_{\mathrm{f}}}$ is the magnetic parameter, ${P}_{\mathrm{r}}=\frac{{\left(\mu {c}_{\mathrm{p}}\right)}_{\mathrm{f}}}{k_{\mathrm{f}}}$ is the Prandtl number, $Ec=\frac{{\left(r{\upsilon}_{\mathrm{f}}\right)}^2}{a^4\left({T}_1-{T}_2\right){\left({c}_{\mathrm{p}}\right)}_{\mathrm{f}}}$ is the Eckert number and *Br* = Pr. *Ec* is the Brinkman number.

It is worth-mentioning here that the continuity Eq. (1) is identically satisfied, that is, the proposed velocity is compatible with Eq.(1) and, thus, represents possible fluid motion.

Finally, we set $f=\frac{F}{\mathrm{Re}}$, and consider the case (following Kashif et al. [15]), we take *Aa*′(*t*) = *υ*_w_, and then the permeable Reynolds number becomes $\mathrm{R}\mathrm{e}=\frac{a(t){\upsilon}_{\mathrm{w}}}{\upsilon_{\mathrm{f}}}$. When *α* is a constant *f* = *f* (*η*), *θ* = *θ*(*η*) and *χ* = *χ*(*η*) which leads to *χ*_*t*_ = 0, *θ*_*t*_ = 0, and *f*_*ηη t*_ = 0. Thus, we have

19 $$ \frac{\upsilon_{nf}}{\upsilon_f}{f}_{\eta \eta \eta \eta}+\alpha \left(3{f}_{\eta \eta }+\eta {f}_{\eta \eta \eta}\right)-2\mathrm{R}\mathrm{e}f{f}_{\eta \eta \eta }-\frac{\rho_f}{\rho_{nf}}M{f}_{\eta \eta }=0, $$

20 $$ \left(1+4Tr/3\right){\theta}_{\eta \eta }+\frac{\upsilon_{\mathrm{f}}}{\alpha_{\mathrm{nf}}}\left(\eta \alpha -2\mathrm{R}\mathrm{e}f\right){\theta}_{\eta }+{\mathrm{Re}}^2\;\left({\left(1-\phi \right)}^{-2.5}{f_{\eta \eta}}^2\right)Br\;\left(\frac{k_{\mathrm{f}}}{k_{\mathrm{nf}}}\right)=0, $$

21 $$ {\chi}_{\eta \eta }+Sc\;\left(\eta \alpha -2\mathrm{R}\mathrm{e}f\right){\chi}_{\eta }=0, $$

22 $$ \begin{array}{l}f=-1;\;{f}_{\eta }=0,\;\mathrm{at}\;\eta =-1\;\mathrm{when}\kern0.24em \theta =1\;\mathrm{and}\;\chi =1,\\ {}f=1;\;{f}_{\eta }=0,\;\mathrm{at}\;\eta =1\;\mathrm{when}\kern0.24em \theta =0\;\mathrm{and}\;\chi =0.\end{array} $$

The physical quantities of engineering applications are the skin friction coefficient *C*_f_, the Nusselt number *Nu*, and the Sherwood number *Sh*, which can be written as

$$ {C}_{\mathrm{f}}=\frac{2{\tau}_{\mathrm{rz}}}{\rho_{\mathrm{f}}{u}^2},\kern0.24em  Nu=\frac{r{q}_{\mathrm{w}}}{K_{\mathrm{f}}\left({T}_1-{T}_2\right)},\;Sh=\frac{r{q}_{\mathrm{m}}}{D\left({C}_1-{C}_2\right)}, $$

where *τ*_rz_ is the disk radial shear stress and *q*_w_ and *q*_m_ are the wall heat and mass flux of the lower disk, respectively. These parameters are given by

23 $$ {\tau}_{\mathrm{r}\mathrm{z}}={\mu}_{\mathrm{nf}}{\left(\frac{\partial u}{\partial z}\right)}_{z=-1},\ {q}_{\mathrm{w}}={q}_{\mathrm{r}}-{K}_{\mathrm{nf}}{\left(\frac{\partial T}{\partial z}\right)}_{z=-1},\ {q}_{\mathrm{m}}=-D{\left(\frac{\partial C}{\partial z}\right)}_{z=-1}. $$

## Numerical Solution

A numerical technique known as the “shooting method” based on Runge-Kutta fourth order is applied and is bound to the system of nonlinear coupled Eqs. (20)–(22) with boundary conditions Eq. (23). Before applying the numerical method, we convert the governing DEs into a system of first-order ordinary differential equations (ODEs).

A common methodology is to compile the nonlinear ODEs as a system of first order initial value problems as follows:

Put *f*′ = *a*, *f*″ = *b*, *f*‴ = *c*, *θ*′ = *d*, *χ*′ = *e*, in Eqs. (20)–(22), then we have *f*′ = *a*, *a*′ = *b*, *b*′ = *c*,

and

24 $$ \left\{\begin{array}{l}{c}^{\prime }=-\frac{\upsilon_{\mathrm{f}}}{\upsilon_{\mathrm{nf}}}\left[\alpha \left(3b+\eta c\right)-\left( Mb+2\mathrm{R}\mathrm{e}\right) fc\right]\\ {}{d}^{\prime }=-{\left(1+4Tr/3\right)}^{-1}\left[\left(\eta \alpha -2\mathrm{R}\mathrm{e}f\right)c+{\mathrm{Re}}^2{\left(1-\phi \right)}^{-2.5}.{b}^2\Big)Br.\frac{K_{\mathrm{f}}}{K_{\mathrm{nf}}}\right]\;\\ {}{e}^{\prime }=-Sc\left(\eta \alpha -2\mathrm{R}\mathrm{e}f\right)e\end{array}\right\}. $$

With the following obligatory boundary conditions:

25 $$ f\left(-1\right)=-1,a\left(-1\right)=-1,\theta \left(-1\right)=1,\chi \left(-1\right)=1,\;b\left(-1\right)=\varTheta 1,c\left(-1\right)=\varTheta 2,d\left(-1\right)=\varTheta 3,e\left(-1\right)=\varTheta 4. $$

Here, *Θ*1, *Θ*2, *Θ*3, and *Θ*4 are missing initial conditions. Therefore, at this stage we apply a shooting method which is an accurate and effective way to determine the unknown initial conditions with the least computation. It is imperative to note that the missing initial conditions are computed until the solution satisfies the boundary conditions *f*(1) = 1, *a*(1) = 0, *θ*(1) = 0, *χ*(1) = 0.

## Results and Discussion

Physical quantities we take into account are the skin friction coefficient, the heat and mass transfer rates at the lower disk which are proportionate to (1 − *ϕ*)^− 2.5^|*f* ' ' (−1)|, $\left(1+\frac{4Tr}{3}\right)\frac{K_{\mathrm{nf}}}{K_{\mathrm{f}}}\left|{\theta}^{\prime}\left(-1\right)\right|$ and |*χ* ' (−1)|, respectively. The parameters that govern this study are as follows: Re is the permeable Reynolds number, *ϕ* is the nanoparticle volume fraction parameter, *M* is the magnetic parameter, *α* is the wall expansion ratio, *Br* is the Brinkman number, *Sc* is the Schmidt number, and *Tr* is the thermal radiation parameter. Note that *α* < 0 or *α* > 0 according to the case when the disks are contracting or relaxing, while Re < 0 for suction and Re > 0 for injection.

In Table 1, we indicate how the abovementioned parameters affect shear stress, heat, and mass transfer rate at the lower disk, whether the disks are relaxing or contracting. For the relaxing case, *M* escalates the shear stress along with the heat transfer rate for suction as well as for injection, but *M* drops the mass transfer rate in the case of suction and rises in the case of injection. However, in the contracting case, suction drops the heat and mass transfer. But heat transfer rate significantly escalates for two cases of the permeable Reynolds number Re. Table 2 explains the behavior of the heat and mass transfer rate under the effect of thermal radiation in the presence of nanoparticles. Thermal radiative heat flux reduces the heat transfer rate but the opposite tendency is seen for mass transfer rate.

**Table 2:** Effect of *Tr* on heat and mass transfer rate for *Pr* = 6.2, *M* = *Br* = *Re* = 1

*α*	*M*	Re	(1 − ϕ)^− 2.5^|*f* ' ' (−1)|	$\left(1+\frac{4Tr}{3}\right)\frac{K_{\mathrm{nf}}}{K_{\mathrm{f}}}\left|{\theta}^{\prime}\left(-1\right)\right|$	|*χ* ' (−1)|
1	1	−2	1.7367	1.1646	0.1347
		−1	1.6334	0.3773	0.2222
		1	2.2353	0.8153	0.5372
		2	4.9848	6.6129	0.7577
	3	−2	1.8443	1.2178	0.1353
		−1	1.8065	0.3895	0.2230
		1	2.7800	0.7700	0.5347
		2	5.5159	6.6559	0.7537
	5	−2	1.9515	1.2731	0.1361
		−1	1.9750	0.4040	0.2237
		1	3.2411	0.7600	0.5326
		2	5.9635	6.7693	0.7504
−1	1	−2	2.7219	2.6761	0.2813
		−1	3.5276	0.7798	0.4696
		1	7.5649	0.9732	0.9592
		2	8.3928	7.2856	1.0967
	3	−2	2.1821	1.6000	0.2405
		−1	3.7173	0.7956	0.4704
		1	7.7960	1.0016	0.9585
		2	7.1262	5.3874	1.0068
	5	−2	1.8227	1.0240	0.2448
		−1	3.8999	0.8133	0.4712
		1	8.0133	1.0304	0.9578
		2	5.7594	3.5199	1.0006
		*Tr*	ϕ	$\left(1+\frac{4Tr}{3}\right)\frac{K_{nf}}{K_f}\left|{\theta}^{\prime}\left(-1\right)\right|$	|*χ* ' (−1)|
		0.1	0	3.8648	0.5343
			0.05	3.7882	0.5357
			0.07	3.7766	0.5363
			0.1	3.7751	0.5372
		0.5	0	2.6586	0.5343
			0.05	2.6138	0.5357
			0.07	2.6081	0.5363
			0.1	2.6003	0.5372
		1	0	1.3910	0.5343
			0.05	0.7962	0.5357
			0.07	0.8011	0.5363
			0.1	0.8153	0.5372

**Fig. 2:**
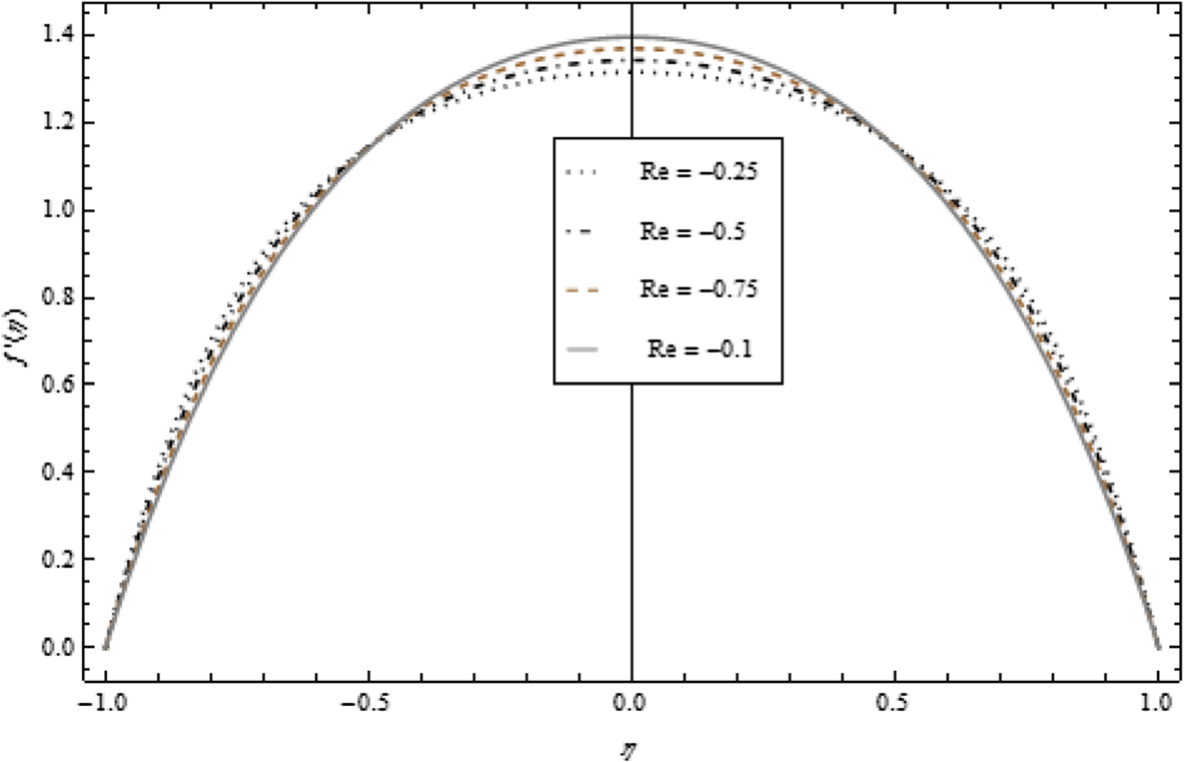
Velocity profile under the influence of Re < 0 for {*α* = 1, *M* = *Br* = *Tr* = *Sc* = 1, ϕ = 0.1}

**Fig. 3:**
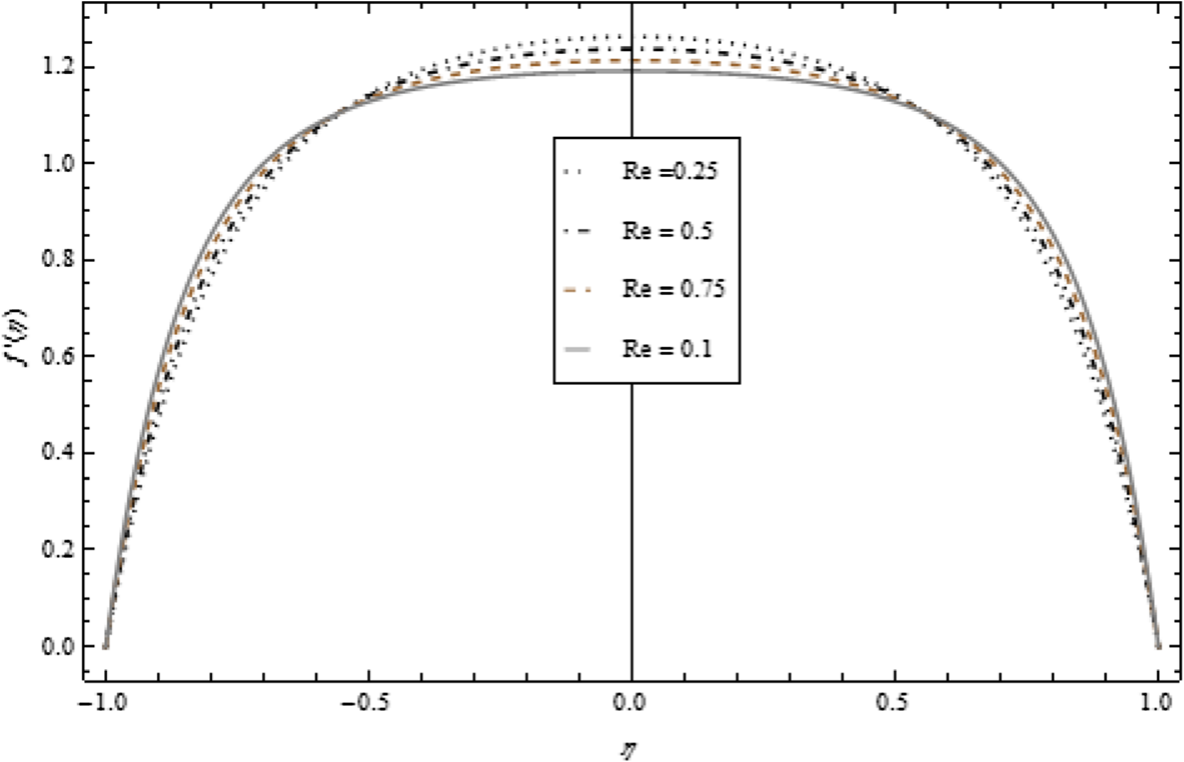
Velocity profile under the influence of Re > 0 for {*α* = 1, *M* = *Br* = *Tr* = *Sc* = 1, ϕ = 0.1}

**Fig. 4:**
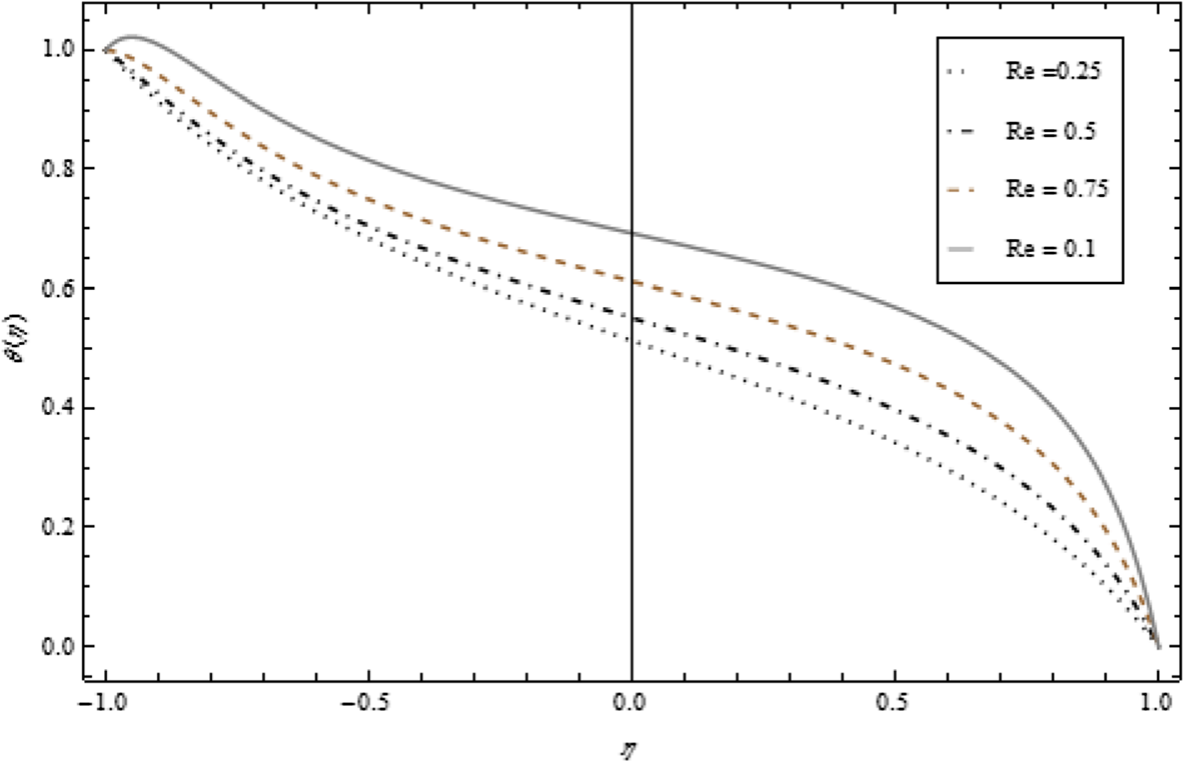
Temperature profile under the influence of Re > 0 for {*α* = 1, *M* = *Br* = *Tr* = *Sc* = 1, ϕ = 0.1}

**Fig. 5:**
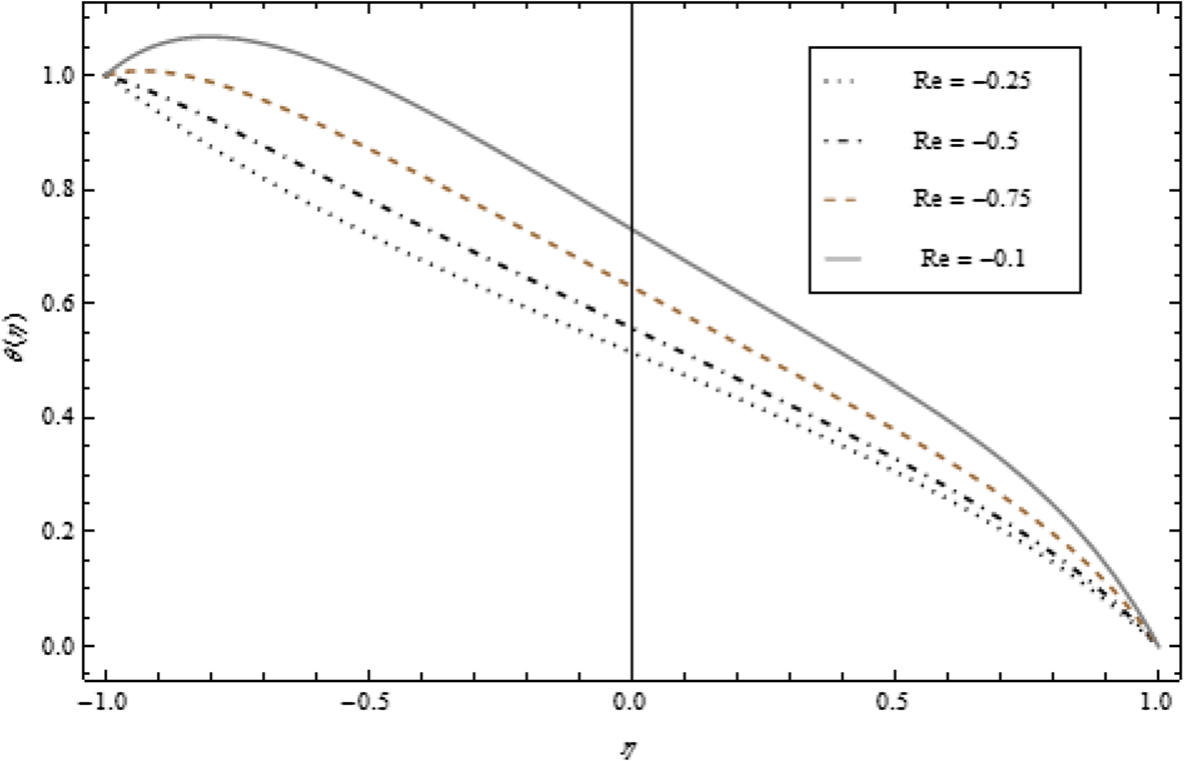
Temperature profile under the influence of Re < 0 for {*α* = 1, *M* = *Br* = *Tr* = *Sc* = 1, ϕ = 0.1}

**Fig. 6:**
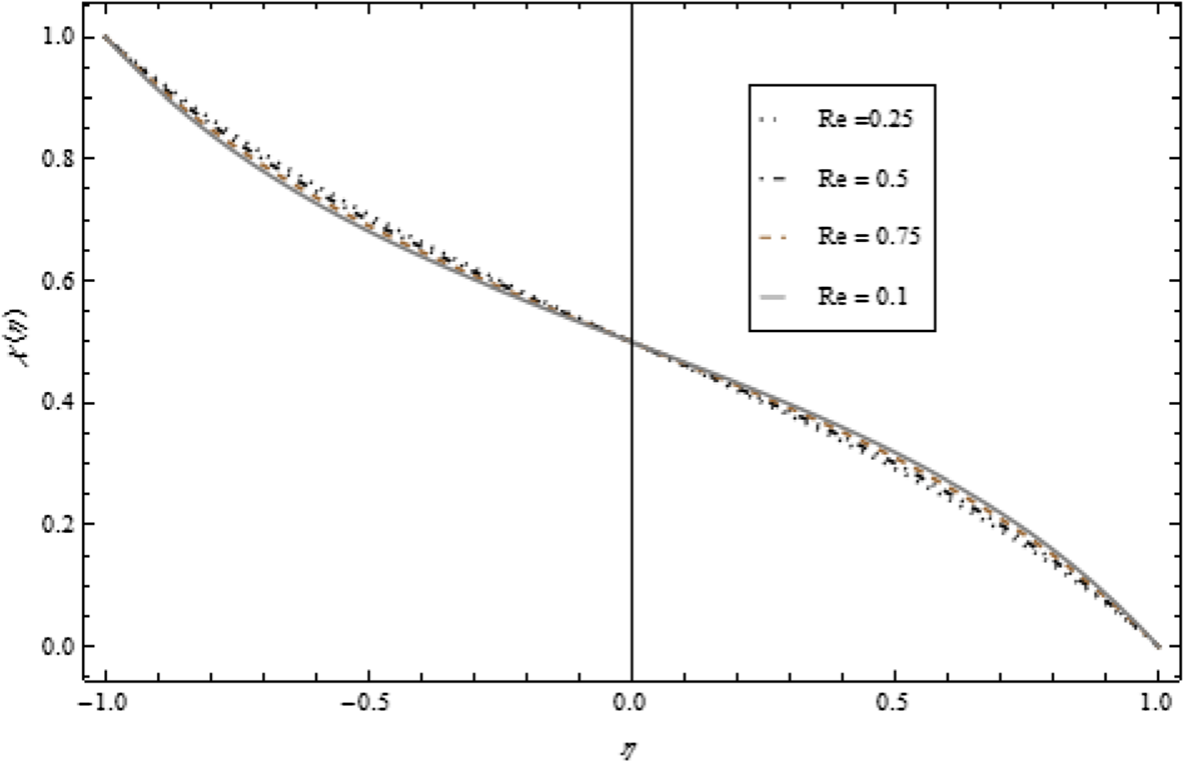
Mass Transfer profile under the influence of Re > 0 for {*α* = 1, *M* = *Br* = *Tr* = *Sc* = 1, ϕ = 0.1}

**Fig. 7:**
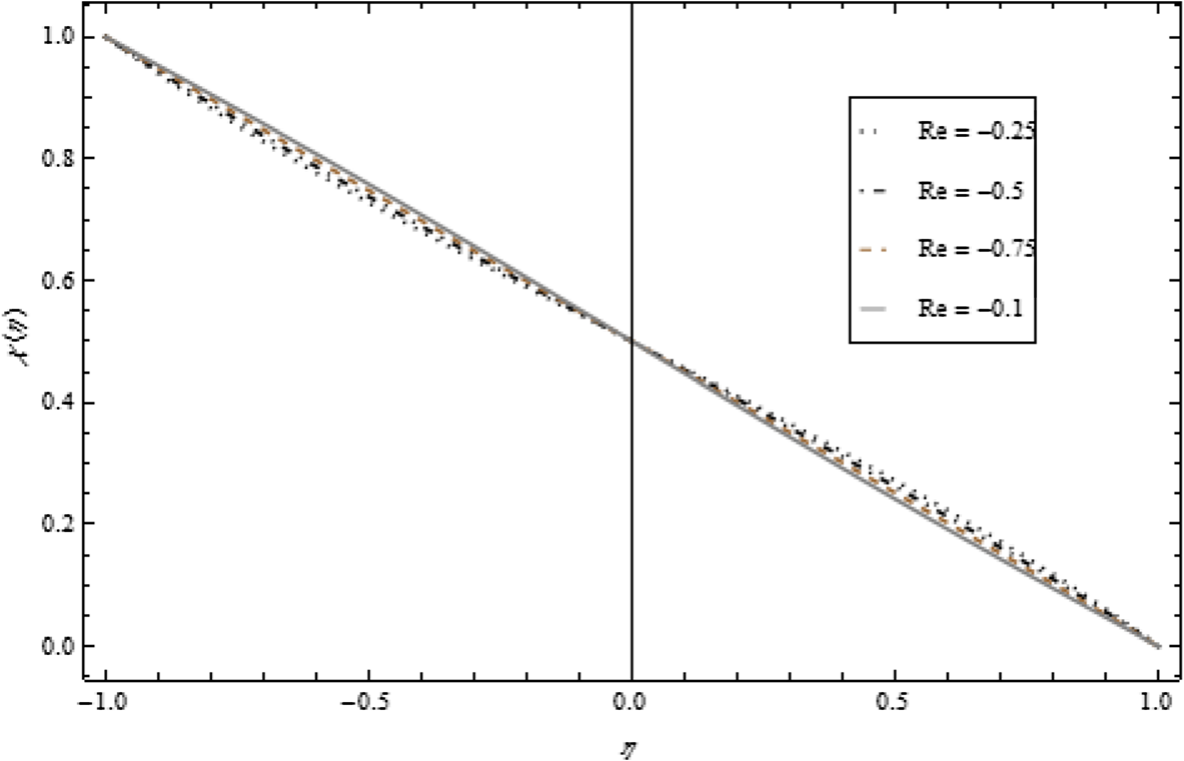
Mass profile under the influence of Re < 0 for {*α* = 1, *M* = *Br* = *Tr* = *Sc* = 1, ϕ = 0.1}

Figures 2, 3, 4, 5, 6, and 7 depict the behavior of Re on velocity, heat, and mass transfer profiles. In the case of suction, increasing behavior is observed in the center of the disks and decreasing tendency is viewed nearby the lower and upper disks as demonstrated in Fig. 2.

**Fig. 8:**
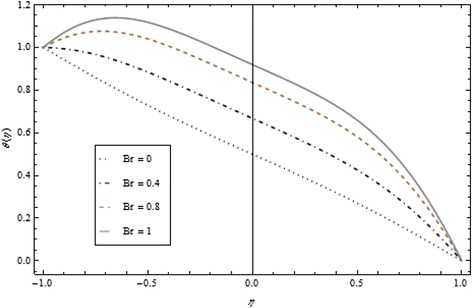
Temperature profile under the influence of *Br* for {*α* = 1, *M* = *Tr* = *Sc* = 1, ϕ = 0.1}

**Fig. 9:**
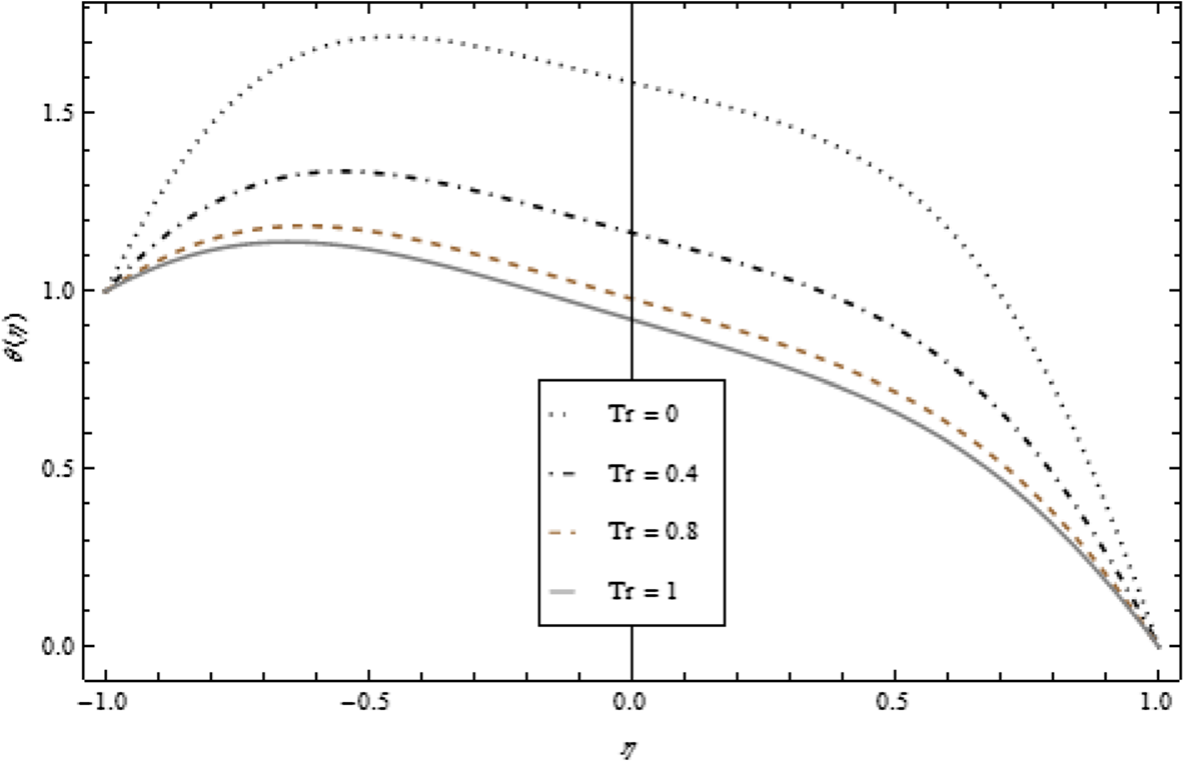
Temperature profile under the influence of *Tr* for {*α* = 1, *M* = *Br* = *Sc* = 1, ϕ = 0.1}

**Fig. 10:**
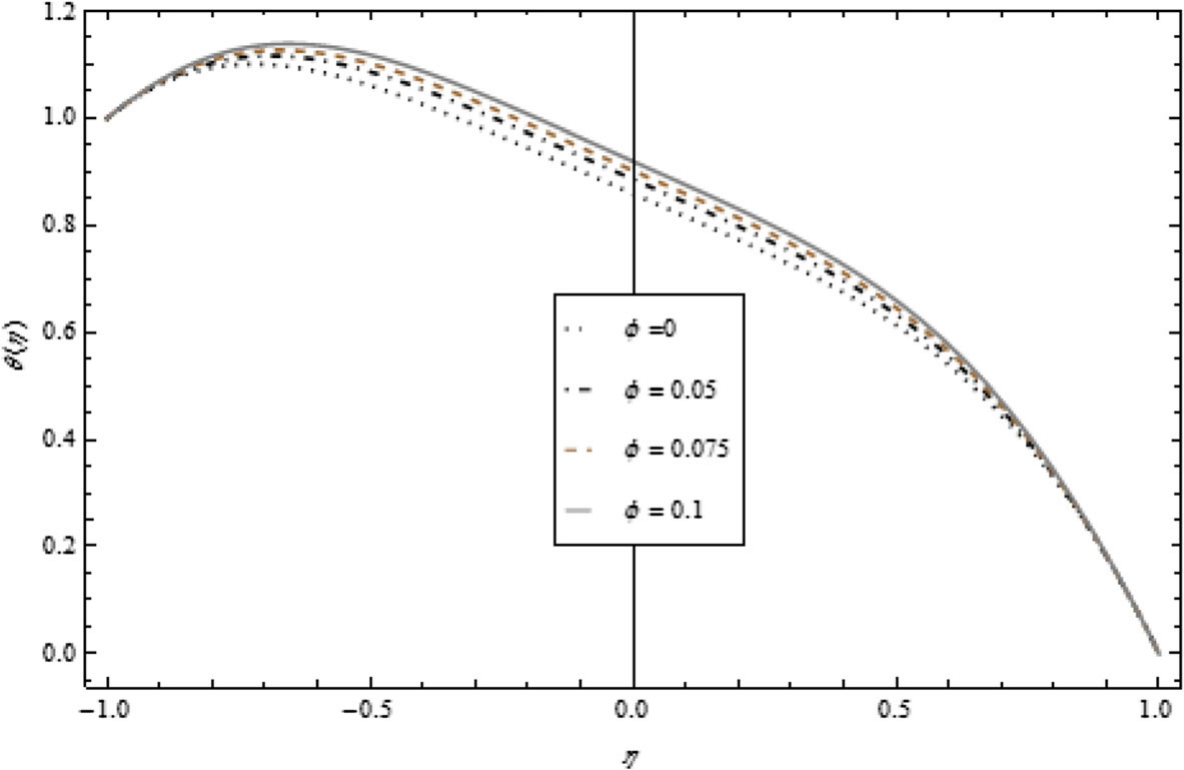
Temperature profile under the influence of ϕ for {*α* = 1, *M* = *Br* = *Tr* = *Sc* = 1}

**Fig. 11:**
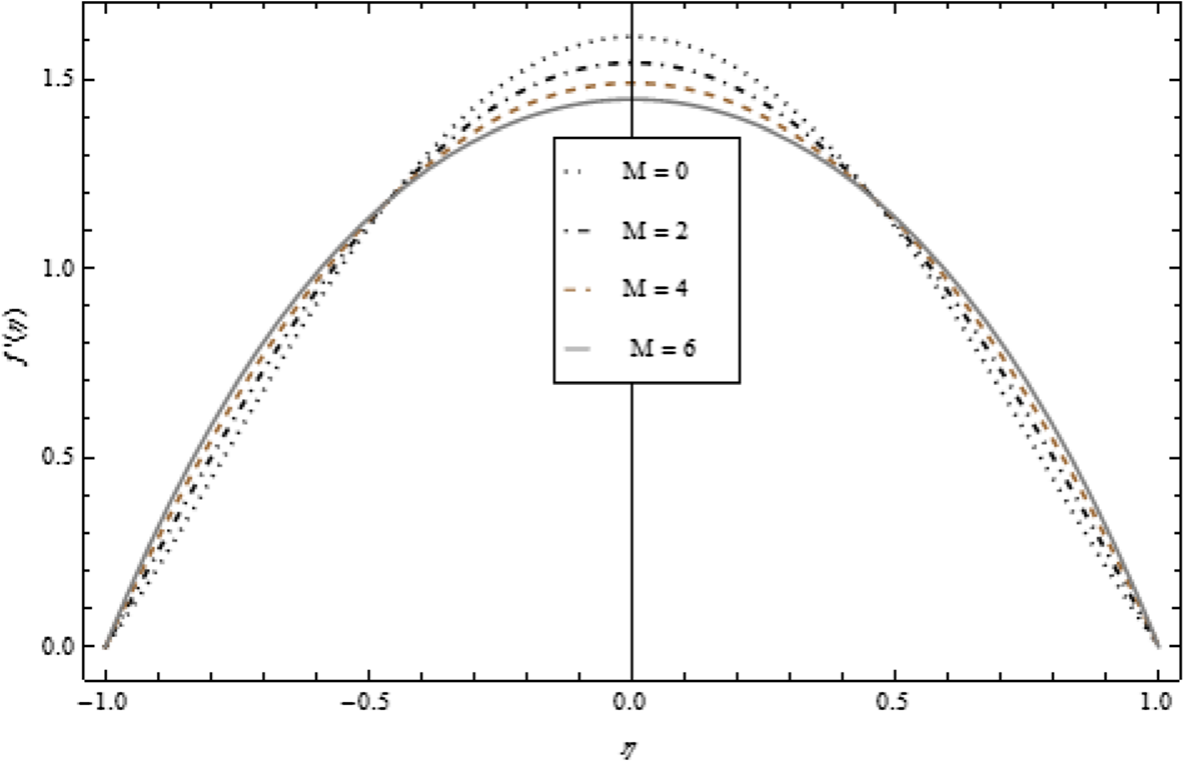
Velocity profile under the influence of *M* for {*α* = 1, *Br* = *Tr* = *Sc* = 1, ϕ = 0.1}

**Fig. 12:**
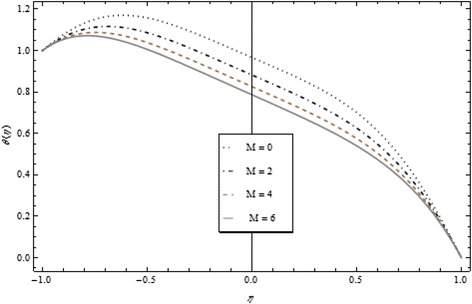
Temperature profile under the influence of *M* for {*α* = 1, *Br* = *Tr* = *Sc* = 1, *ϕ* = 0.1}

**Fig. 13:**
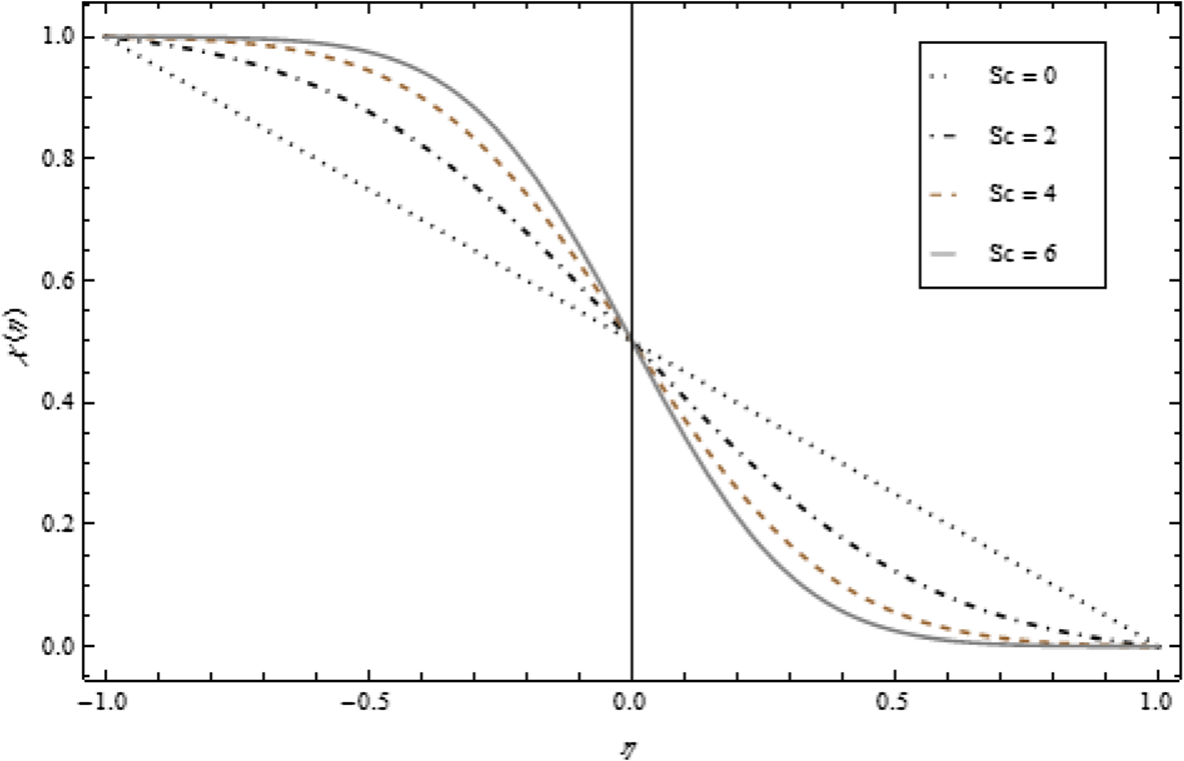
Mass transfer profile under the influence of *Sc* for {*α* = 1, *M* = *Br* = *Tr* = 1, ϕ = 0.1}

**Fig. 14:**
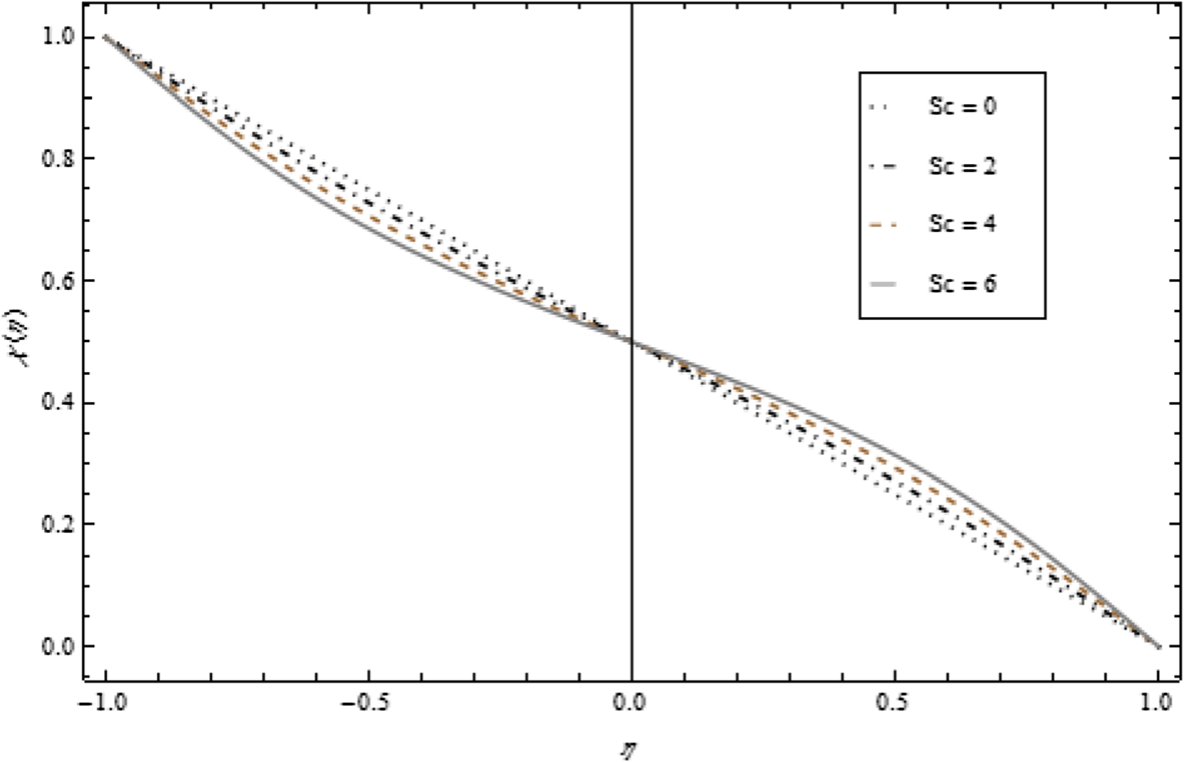
Mass transfer profile under the influence of *Sc* for {*α* = 1, *M* = *Br* = *Tr* = 1, Re = − 1, ϕ = 0.1}

Thickness of the momentum boundary layer is an increasing function of Re < 0. Figure 3 demonstrates quite the opposite trend for the injection case. Heat transfer profiles significantly increase across the whole domain of the disks for suction and injection cases as shown in Figs. 4 and 5. Injection increases the mass transfer profile nearby the upper disk and decreases nearby the lower disk. The reverse tendency is noted in the case of suction as shown in Figs. 6 and 7. Brinkman number *Br* is vital phenomenon for heat conduction in a porous surface and has a considerable effect on heat transfer. Due to the existence of metallic spherical nanoparticles, heat transfer is an increasing function of *Br* and a decreasing function of thermal radiative heat flux with injection as given in Figs. 8 and 9. Heat transfer escalates with increase in nanoparticles volume fraction as described in Fig. 10. The external magnetic field has a tendency to reduce velocity in the center of the two disks. So for this area, the magnetic field behaves like a drag force which is known as the Lorentz force. This force ultimately reduces the fluid velocity as well as temperature profile as exhibited in Figs. 11 and 12. The thickness of the momentum boundary layer is also a decreasing function of *M*. Figures 13 and 14 demonstrate the behavior of mass transfer profile under the effect of *Sc* the Schmidt number with injection and suction effects, respectively. Basically, *Sc* is the ratio of kinematic viscosity to mass diffusivity coefficient, *Sc* is an increasing function, and then dominant kinematic viscosity function has a significant effect on mass transfer profile. Decreasing function is observed near the upper disk and vice versa exists near the lower disk for the injection case as shown in Fig. 13. For the suction case, the opposite trend is observed in Fig. 14.

## Conclusions

In this paper, we undertook a numerical study to explore the mechanism which explains the effects of governing parameters on flow and heat transfer features of laminar, incompressible, unsteady, two-dimensional flow of a nanofluid, which is water-based and contains gold spherical nanoparticles, between two porous coaxial disks that are moving orthogonally. In the case of expanding disks (*α* > 0), heat transfer rate and shear stress at the lower disk escalate with *M* and Re, whereas heat transfer rate falls with *ϕ* and *Tr*. Moreover, mass transfer rate decreased in the case of suction and increased in the case of injection. As far as contracting disks (*α* < 0) are concerned, shear stress at the disks escalates with *M* and *α*; however, a reverse impact is found for *ϕ* and *R*. Furthermore, it is concluded that heat transfer rate rises with *M*, *R*, *α*, and *ϕ*.

## Abbreviations

DEs: Differential equations
MHD: Magnetohydrodynamic
ODEs: Ordinary differential equations
